# The Diversity of Fungal Endophytes from Wild Grape *Vitis amurensis* Rupr

**DOI:** 10.3390/plants11212897

**Published:** 2022-10-28

**Authors:** Olga A. Aleynova, Nikolay N. Nityagovsky, Andrey R. Suprun, Alexey A. Ananev, Alexandra S. Dubrovina, Konstantin V. Kiselev

**Affiliations:** Laboratory of Biotechnology, Federal Scientific Center of the East Asia Terrestrial Biodiversity, Far Eastern Branch of the Russian Academy of Sciences, 690022 Vladivostok, Russia

**Keywords:** biodiversity, endophytes, fungi, fungal diversity, fungal microbiome, grapevine, ITS1, next-generation sequencing, *Alternaria* spp., *Cladosporium* spp., *Vitis amurensis*

## Abstract

Grapevine endophytic fungi have great potential for application in agriculture and represent an important source of various compounds with valuable biological activities. Wild grapevine is known to host a great number of rare and unidentified endophytes and may represent a rich repository of potential vineyard biocontrol agents. This investigation aimed to study the fungal endophytic community of wild grape *Vitis amurensis* Rupr. using a cultivation-dependent (fungi sowing) and a cultivation-independent (next-generation sequencing, NGS) approach. A comprehensive analysis of the endophytic fungal community in different organs of *V. amurensis* and under different environmental conditions has been performed. According to the NGS analysis, 12 taxa of class level were presented in different grapevine organs (stem, leaf, berry, seed). Among the 12 taxa, sequences of two fungal classes were the most represented: *Dothideomycetes*—60% and *Tremellomycetes*—33%. The top five taxa included *Vishniacozyma*, *Aureobasidiaceae*, *Cladosporium*, *Septoria* and *Papiliotrema*. The highest number of fungal isolates and sequences were detected in the grape leaves. The present data also revealed that lower temperatures and increased precipitation favored the number and diversity of endophytic fungi in the wild Amur grape. The number of fungi recovered from grape tissues in autumn was two times higher than in summer. Thus, this study is the first to describe and analyze the biodiversity of the endophytic fungal community in wild grapevine *V. amurensis*.

## 1. Introduction

Plant endophytes are mainly represented by bacteria and fungi, while archaebacteria, algae, protozoa and nematodes are rarely detected as endophytes [1,2]. Endophytes are distributed asymptomatically in plant tissues such as roots, stems, leaves, seeds and fruits [3,4]. Growing evidence indicates that endophytic associations can be important for plant fitness and crop productivity [5,6]. Subsequently, considerable evidence indicated that endophytic associations are important for plant nutrient acquisition [7], immune system [8], disease suppression [9], tolerance to abiotic stresses [10], and phytohormone production [11,12].

Endophytic fungi attract special attention among all endophytes in terms of application in agriculture. Currently, many reports have focused on endophytic fungi as a rich source of valuable bioactive compounds, while other reports focused on endophytic fungi as biocontrol agents [12]. For example, the fungal endophytes of cork oak *Simplicillium aogashimaense, Fimetariella rabenhorstii, Chaetomium* sp. and *Alternaria alternata* revealed a high potential to inhibit the growth of pathogenic fungi *Biscogniauxia mediterranea* and *Diplodia corticola* that cause great damage to forestry [13]. Endophytic fungi of maize *Epicoccum* and *Sordaria* and wheat endophytic *Trichoderma* strains showed antipathogenic activity against the widespread pathogen *Fusarium graminearum* [14,15].

Grapes are one of the most demanded and economically important agricultural crops in the world. It is known that many endophytic grape fungi possess a considerable potential for reducing the colonization of pathogenic microorganisms that cause grape diseases. The endophytic fungus *Trichoderma* sp. T154 of *Vitis vinifera* L. reduced pathogenic colonization of the esca-related pathogen (*Phaeoacremonium aleophilum*) infecting the same niches [16]. Also, grapevine fungal endophytes are known to alter the profile of produced secondary metabolites in plant hosts. For example, a recent study demonstrated that exposure of grapevine cells to endophytic fungi *Alternaria alternate* and *Epicoccum nigrum* and a dual culture system differentially affected total anthocyanin concentrations and phenylalanine ammonia lyase activities [17]. Cocultivation of grape cells with endophytic fungi *Biscogniauxia* sp., *Cladosporium* sp., *Didymella* sp. 1, and *Didymella* sp. 2 significantly increased the content of stilbenes [18].

In addition, a number of researchers reported that fungal endophytes of grapevine *V. vinifera* contain resveratrol [19,20,21]. The fungal isolates producing resveratrol belonged to seven genera: *Botryosphaeria*, *Penicillium*, *Cephalosporium*, *Aspergillus*, *Geotrichum*, *Mucor* and *Alternaria* [19]. It has also been shown that the ability to produce resveratrol decreased or was completely lost in most endophytes after three rounds of subcultivation. Only the strain *Alternaria* sp. MG1 (isolated *V. vinifera* cv. Merlot) exhibited a stable and high ability to produce resveratrol in all subcultures [19]. In addition, it has been shown that endophytic fungi *Arcopilus aureus* and *Xylaria psidii* isolated from *V. vinifera* grapes contained resveratrol and its derivatives [20,21].

Previously, it has been found that the diversity of endophyte species was far higher for wild grapevine *V. vinifera* than for *V. vinifera* grown in conventional vineyards [22]. In addition, the wild, conventional, and organically grown *Vitis* cultivars demonstrated distinctive communities of fungal endophytes with low species overlap [22]. Wild *V. vinifera* supported a greater number of rare and unidentified endophytes and may represent a rich repository of potential vineyard biocontrol agents [22]. In support of this assumption, the endophytic fungus *Albifimbria verrucaria* isolated from another wild grape species *Vitis amurensis* Rupr. was active against *Botrytis cinerea* causing gray mold disease in grapes [23]. In addition, the high endophyte diversity of wild grapevines could also contribute to their high adaptive potential and high stress resistance. Therefore, studying endophytic communities of wild grapes is of considerable interest. Wild Amur grapevine *V.* *amurensis* is known as a highly resistant species to such widespread grapevine diseases as powdery mildew [24], grape white rot, and anthracnose [25].

The current study focused on studying the fungal endophytic community of *V. amurensis* and aimed to investigate the diversity of fungal endophytes colonizing the stems, leaves, berries and seeds of wild grapevine *V. amurensis* using next generation sequencing (NGS) and cultivation-dependent approaches (fungal sowing). This investigation aimed to isolate and characterize fungal endophytes from *V. amurensis*, since this plant species may represent a rich source of vineyard biocontrol agents.

## 2. Results

### 2.1. The Biodiversity of Fungal Endophytes Inhabiting Different Organs of V. amurensis

**Figure 1:**
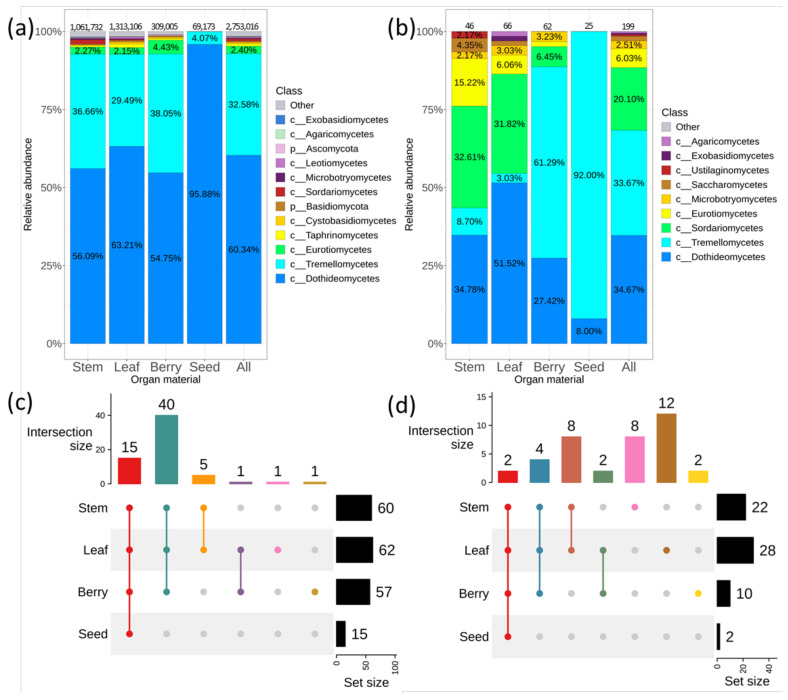
Comparative analysis of the fungal endophytic community composition in different organs of wild grape *Vitis amurensis* according to the cultivation-independent (next-generation sequencing (NGS)) and the cultivation-dependent (fungal sowing (Sow)) approaches. The composition of endophytic fungi of *V. amurensis* depends on the plant organ: (**a**) Class level taxonomical bar plots for the fungal endophytic community in stem, leaf, berry, seed, and the sum of data for all *V. amurensis* organs obtained using NGS; (**b**) Class level taxonomical bar plots for the fungal endophytic community in stem, leaf, berry, seed, and the sum of data for all *V. amurensis* organs obtained by fungal sowing; (**c**) Genus level UpSet diagrams depicting overlapping taxa of NGS in leaf, stem, berry, and seed; (**d**) Genus level UpSet diagrams depicting overlapping taxa of fungal sow in leaf, stem, berry, and seed. Taxa were filtered based on relative abundance of >0.1% for each biocompartment. Taxa of relative abundance of <0.1% were removed from UpSet diagram. Number of sequences or strains (for sow) are shown above taxonomical bar plots.

Next-generation sequencing (NGS) produced a total of 3,559,302 paired reads. After bioinformatic quality control procedures a total of 2,753,016 sequences in 22 samples were identified which belong to 64 taxa of genus level. According to the analysis, 12 taxa of class level were present in different grapevine organs (stem, leaf, berry, seed) with the relative representation above 0.1%. Among the 12 taxa, sequences of two fungal classes were the most represented: *Dothideomycetes—*60% and *Tremellomycetes—*33% (Figure 1a). Fungal endophytes were richer in leaves and stems (1,313,106 and 1,061,732 sequences) than in berries and seeds (309,005 and 69,173 sequences). The fungal taxonomic diversity at genus level in the leaves, stem and berries of wild grapes was much higher than in the seeds (Figure 1c and Figure S1c). The beta diversity data showed diffuse clustering and no significant difference between organ samples performed by PERMANOVA test (Supporting Information 2).

A cultivation-dependent approach (fungal sowing) was also applied to analyze fungal endophytic community in different part of *V. amurensis*. A total of 199 strains were isolated. These strains were divided into nine classes of fungi. *Dothideomycetes*, *Tremellomycetes*, *Sordariomycetes* were the predominant classes (Figure 1b). In addition, this analysis detected *Eurotiomycetes—*6%, *Microbotryomycetes*—2%, *Saccharomycetes*—1.4%, and *Ustilaginomycetes*, *Exobasidiomycetes*, and *Agaricomycetes—*less than 1% (Figure 1b). Also, the biodiversity of fungal endophytes in the leaves was higher than in other parts of *V. amurensis* (Figure 1d). According to the cultivation-dependent approach, 12 unique genera were specific to the leaves of *V. amurensis* (Figure 1d). Thus, the cultivation-dependent approach generally confirmed the data of the metagenomic analysis. Notably, a total of 22 common taxa of endophytic fungi were detected by both the cultivation-dependent and cultivation-independent approaches, while 16 unique taxa were detected by the cultivation-dependent method and 41 unique taxa—by NGS (Figure S2).

**Figure 2:**
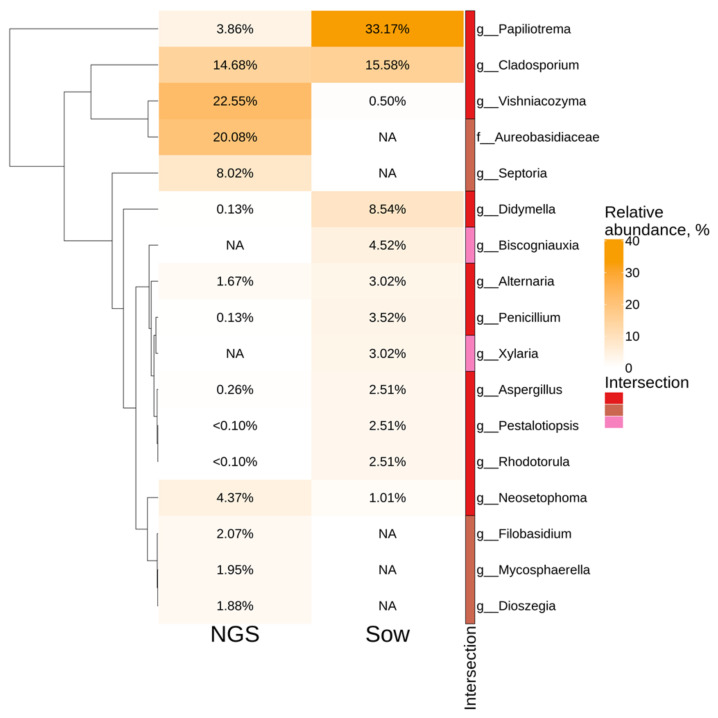
Genus level relative abundance heat maps of significant taxa in *Vitis amurensis* according to the cultivation-independent (next-generation sequencing (NGS)) and cultivation-dependent (fungal sowing (Sow)) methods. The top ten most abundant taxa from each factor are displayed. White squares (NA) represent the absence of taxa. The intersection selection was made based on the UpSet diagram in Supporting Information 3.

The top six taxa according to the NGS data analysis were *Vishniacozyma* (22.5%), *Aureobasidiaceae* (20%), *Cladosporium* (14.7%), *Septoria* (8%), *Neosetophoma* (4.4%) and *Papiliotrema* (3.9%) (Figure 2). According to the cultivation-dependent method, the predominant fungal genera were *Papiliotrema* (33%), *Cladosporium* (16%), and *Didymella* (8.5%) (Figure 2). The *Aureobasidiaceae*, *Septoria*, *Filobasidium*, *Mycosphaerella* and *Dioszegia* taxa were discovered using the NGS method, while they were not detected after fungal sowing (Figure 2).

**Figure 3:**
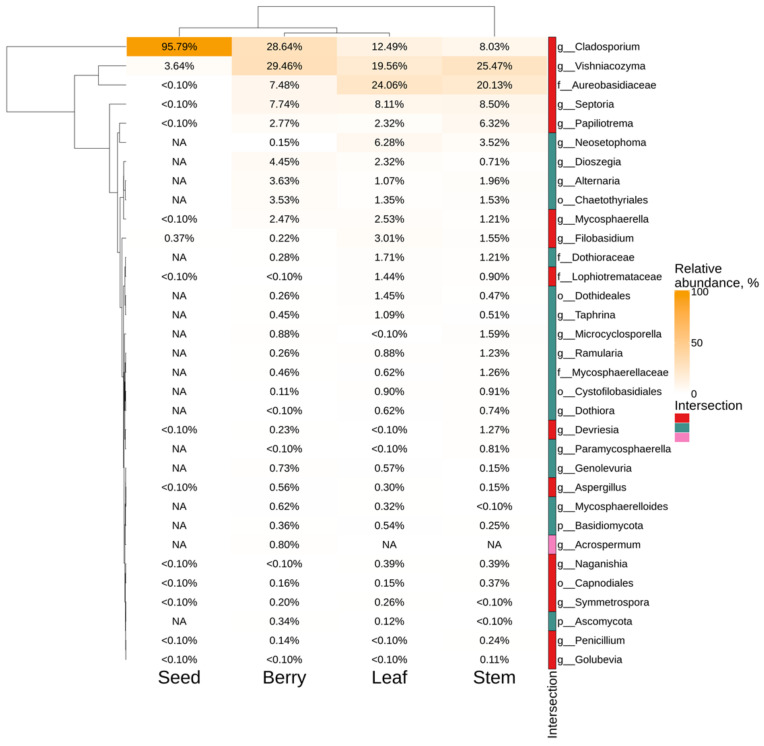
Genus level relative endophytic fungi abundance heat maps of significant taxa according to the next-generation sequencing (NGS) in different organs (seed, berry, leaf, and stem) of *Vitis amurensis*. The top 20 most abundant taxa from each factor are displayed. White squares (NA) represent the absence of taxa. The intersection selection was made based on the Figure 1c.

The most common taxa for the leaves, stems and berries of *V. amurensis* were *Vishniacozyma*, *Cladosporium*, *Aureobasidiaceae* and *Septoria*, while the most dominant genus for the seeds was represented by only *Cladosporium* (95.8%) (Figure 3). The genus *Acrospermum* was representative only for berries of wild grapevine *V. amurensis* (Figure 3).

### 2.2. Differences in the Biodiversity of Fungal Endophytes of V. amurensis Depending on the Year of Tissue Collection 

For the cultivation-dependent approach (fungal sowing), plant material was selected from 2019 to 2021. The material was collected at approximately the same time periods and weather conditions. However, the average weather conditions in the summer–autumn period varied significantly depending on the year (Table 1).

**Figure 4:**
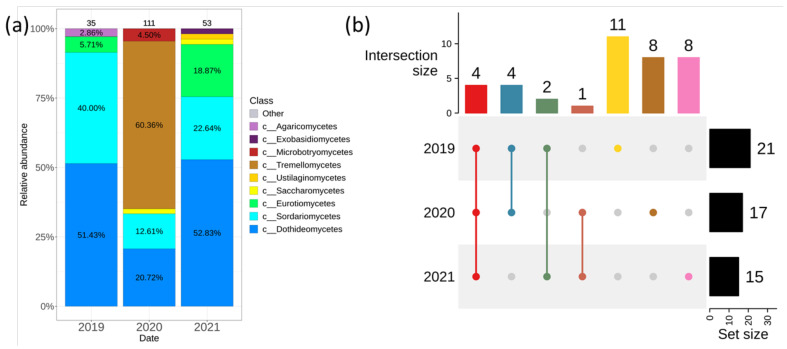
Composition of endophytic fungal community in wild grape *Vitis amurensis* depending on the year of material collection according to the cultivation-dependent (fungal sowing (Sow)) approach. (**a**) Class level taxonomical bar plots for the fungal community composition in 2019–2021; (**b**) Genus level UpSet diagrams depicting overlapping taxa of sow Taxa were filtered based on relative abundance of >0.1% for each biocompartment. Number of colonies are shown above taxonomical bar plots.

In 2019, we isolated and identified 35 isolates, in 2020—111 isolates and in 2021—53 isolates (Figure 4). The predominant classes of endophytic fungi were *Dothideomycetes*, *Sordariomycetes*, *Eurotiomycetes* in all years, except for 2020. 

*Tremellomycetes* was the predominant class in 2020 (Figure 4a). The generic biodiversity was the richest in 2019 (21 genera), and there were 11 unique genera (Figure 4b). Only one major common genus *Cladosporium* was detected for every year of the fungal seeding (Figure 4b and Figure S4b).

### 2.3. Seasonal Variations in the Composition of Endophytic Fungal Community of V. amurensis 

**Figure 5:**
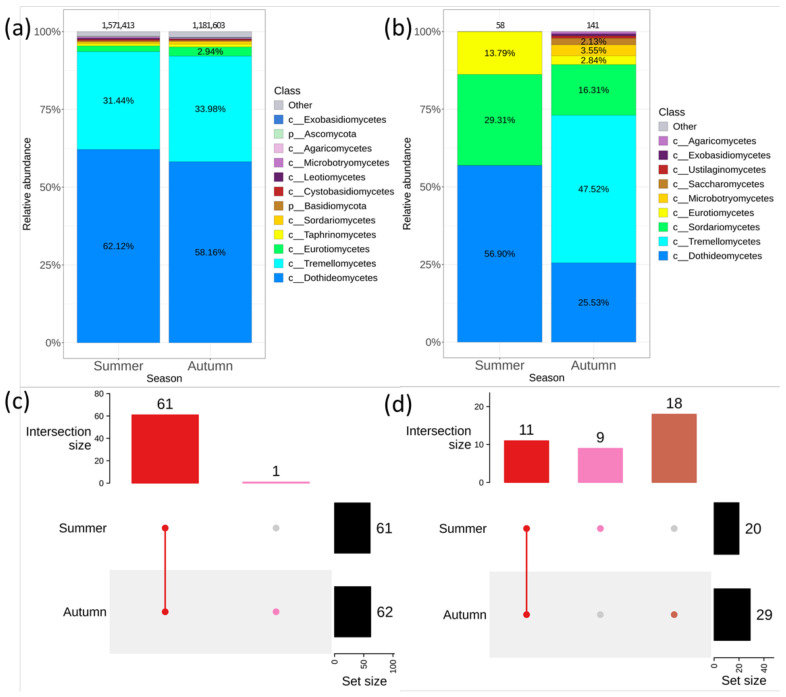
Composition of endophytic fungal community in wild grape *Vitis amurensis* depends on the season of material collection: (**a**) Class level taxonomical bar plots for the fungal community analyzed by the cultivation-independent approach (next-generation sequencing (NGS)) in summer and autumn; (**b**) Class level taxonomical bar plots for the fungal community analyzed by the cultivation-dependent approach in summer and autumn; (**c**) Genus level UpSet diagrams depicting overlapping taxa of NGS in autumn and summer; (**d**) Genus level UpSet diagrams depicting overlapping taxa of fungal sow in summer and autumn. Taxa were filtered based on relative abundance of >0.1% for each biocompartment. Taxa on relative abundance of <0.1% were removed from UpSet diagram. Number of sequences or strains are shown above taxonomical bar plots.

The biodiversity profile of endophytic fungi in *V. amurensis* depending on the collection season was also studied. The leaves and stems of *V. amurensis* were collected in the first half of July and in the second half of September. 1,571,413 sequences were obtained in the summer and 1,181,603 sequences—in the autumn of 2021 using the cultivation-independent approach. The percentage distribution among the classes of endophytic fungi in summer and autumn was approximately the same (Figure 5a). Only the *Acrospermum* genera was unique for the autumn (Figure 5c and Figure S5d). PERMANOVA test showed significant difference between the summer and autumn samples (R^2^: 15.4%, *p* = 0.018) (Supporting Information 2). The most abundant taxa in autumn samples were *Cladosporium* (27.2%), *Vishniacozyma* (26.6%) and *Septoria* (10.1%), while the most prevalent taxa in summer samples were *Aureobasidiaceae* (30.7%), *Vishniacozyma* (19.5%) and *Neosetophoma* (7.2%) (Figure S5d).

The cultivation-dependent approach resulted in a greater number of fungi strains in autumn (141) than in summer probes (58) (Figure 5b). A total of 11 genera of fungi were common for summer and autumn, while 9 genera were unique for summer and 18—for autumn (Figure 5d and Figure S5c). 

### 2.4. Comparative Analysis of Endophytic Fungal Communities in V. amurensis and V. vinifera

A study of endophytic fungi biodiversity in *V. vinifera* cv. ‘Syrah’ [26] was selected for the comparative analysis of endophytic mycobiomes of *V. amurensis* and *V. vinifera*. The main criteria for the selection of this metagenomic study were the presence of microorganism film removal step in the process of endophyte isolation, different geographical locations, the same fungal *ITS1* rDNA region (*ITS1*–*ITS2*) and Illumina sequencing technology. From the Deyett′s and Rolshausen’s (2020) study, we selected samples belonging to the above-ground parts of *V. vinifera* collected in autumn and carried out a comparative analysis with our autumn samples of *V. amurensis*. The samples used in the comparative analysis are presented in Supporting Information 7. 

**Figure 6:**
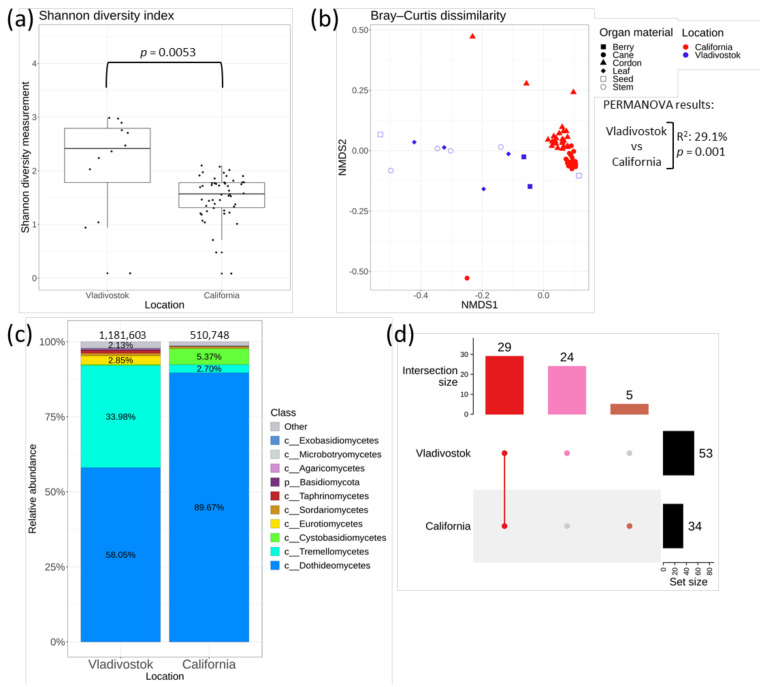
A comparison of endophytic fungal communities of cultivated *Vitis vinifera* from USA (California) with the endophytic community of *Vitis amurensis* from the Russian Far East (Vladivostok) (**a**) Shannon′s alpha diversity boxplot; (**b**) Bray–Curtis beta diversity NMDS plot; (**c**) Class level taxonomical bar plots for the fungal community of *V. vinifera* and *V. amurensis;* (**d**) Genus level UpSet diagrams depicting overlapping taxa in samples. Taxa were filtered based on relative abundance of >0.1% for each biocompartment. Filtered taxa in bar plots were placed in “other” category and removed from UpSet diagram. Number of sequences are shown above taxonomical bar plots.

**Figure 7:**
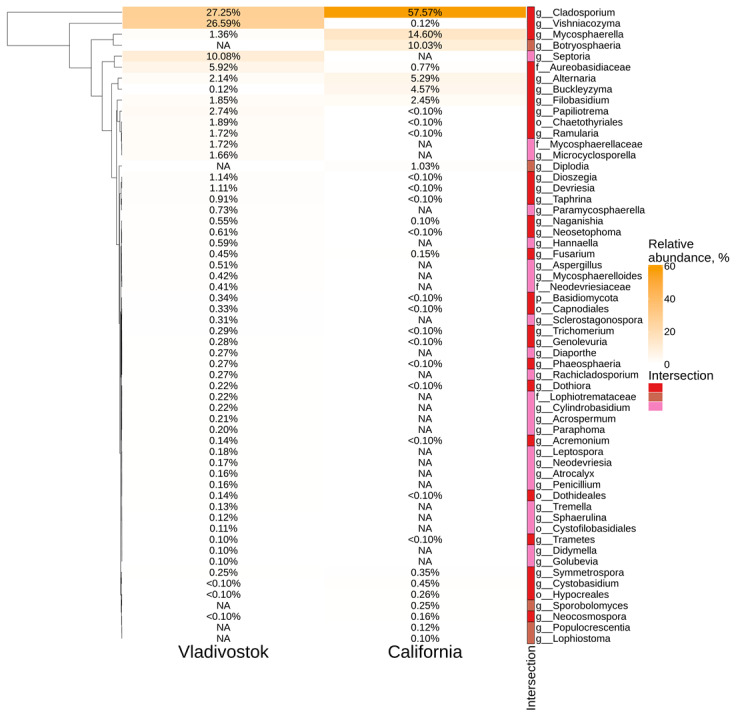
Genus level relative endophytic fungal abundancy heat maps of significant taxa from *Vitis vinifera* (USA, California) and *Vitis amurensis* (Russian Far East, Vladivostok). White squares (NA) represent the absence of taxa. The intersection selection was made based on the Figure 6d.

The amplicon data of each sample site with respect to the location were analyzed. The results for alpha and beta diversity analysis are shown in the Figure 6a,b, respectively. The grape samples collected in Russia (Vladivostok) and USA (California) were statistically different in alpha diversity. Shannon′s diversity index for *V. amurensis* samples was higher in comparison with that for *V. vinifera* samples. The beta diversity results are presented in the nonmetric multidimensional scaling (NMDS) ordination (Figure 6b). NMDS ordination showed that samples from Russia (Vladivostok) and USA (California) located in separate clusters (Figure 6b). The PERMANOVA test demonstrated that the samples collected in Vladivostok and California were significantly different (R^2^: 29.1%, *p* = 0.0053) based on beta diversity (Figure 6b). These results were also supplemented by the UpSet intersection diagram. Autumn samples collected in Vladivostok showed 53 taxa, where 24 taxa were unique. In contrast, California′s samples showed 34 taxa, where only 5 taxa were unique. A total of 29 taxa were common for all samples (Figures 6d and 7).

According to the analysis, the grape endophytic mycobiome is represented by nine main classes of fungi (Figure 6c). Both mycobiomes differed greatly in relative abundance of classes. In *V. amurensis*, the most prevalent classes were *Dothideomycetes* (58%), *Tremellomycetes* (34%) and *Eurotiomycetes* (2.8%), while in *V. vinifera* the most abundant classes were *Dothideomycetes* (89.7%), *Cystobasidiomycetes* (5.4%) and *Tremellomycetes* (2.7%). The top five most abundant taxa of genus level in *V. amurensis* samples were *Cladosporium* (27.2%), *Vishniacozyma* (26.6%), *Septoria* (10.1%), *Aureobasidiaceae* (5.9%) and *Papiliotrema* (2.7%), where genera *Septoria* was unique for these samples. In contrast, *Cladosporium* (57.6%), *Mycosphaerella* (14.6%), *Botryosphaeria* (10%), *Alternaria* (5.3%) and *Buckleyzyma* (4.6%) are the richest taxa in *V. vinifera* samples, in which *Botryosphaeria* was unique for these samples (Figure 7).

## 3. Discussion

Grapevine endophytic fungi have attracted great attention for a variety of reasons, including their antipathogenic properties that have arisen due to competition for space within the host plant [16] and/or due to production of antimicrobial compounds, such as stilbenes [19,21]. Novel methods of disease biocontrol are particularly pertinent for vineyards. Using biological control methods instead of fungicides would also aid in the production of certified organic wines for which there is an increasing demand [27].

It has been earlier shown that endophyte richness and diversity were far higher in wild grapevine from Canada than in conventional vineyards [22]. According to the study, the wild, conventional and organically grown *Vitis* exhibited distinctive communities of fungal endophytes with low species overlap [22].

This study is the first comprehensive effort to characterize a group of endophytic fungi associated with asymptomatic tissue of wild grapevine *V. amurensis* in Russia’s Far East. Thus, wild grapevine *V. amurensis* contained a greater number of rare and unidentified endophytes in comparison with *V. vinifera* and may represent a rich repository of potential vineyard biocontrol agents. The dominant class of the fungal endophytic community in the wild *V. amurensis* was the class *Dothideomycetes* (59%). Also, the main classes of endophytic fungi of *V. amurensis* included *Tremellomycetes* and *Sordariomycetes*. 

The presence of certain fungal taxa in specific grapevine organs probably depends on the content of different nutrients, salts, secondary metabolites, and also on the ability of individual taxa to penetrate into various grape tissues. These factors could be important for growth and development for some fungal endophyte taxa, but this assumption requires further research. *Vishniacozyma*, *Cladosporium*, *Aureobasidiaceae* and *Septoria* were the major genera in all leaves, stems, and berries of *V. amurensis*. Recently, it has been described that *Cladosporium cladosporioides* causes grape fruit rot in Xinjiang, China [28]. Also, the Septoria blotch of spring wheat leaves and ears is considered as one of the most economically significant infections in the Siberian region [29]. Probably, *Cladosporium* sp. and *Septoria* sp. of wild grape *V. amurensis* may occupy a similar ecological niche in a microbiological consortium and act as a biological control of the spread of pathogenic *C. cladosporioides* and *Septoria tritici*. However, this assumption requires further future research. Notably, *Vishniacozyma* was the most abundant genus in Cabernet Sauvignon grapes *V. vinifera* (organic vineyard in Xinjiang) [30]. Thus, the cultivated vines of *V. vinifera* from Xinjiang have similarities in endophytic fungal composition to wild *V. amurensis*, probably due to the close geographical localization.

The results obtained, demonstrate that mycobiota of wild grapevine *V. amurensis* forms a unique composition of endophytic fungi. In addition, the composition and number of endophytic fungi occurring in *V. amurensis* significantly varied depending on plant organs and weather conditions. The analysis of the fungal community in different organs of *V. amurensis* showed that most endophytic fungi inhabited stems and leaves. This conclusion confirmed the previously known information that the endophytes of grapes come from the root system through conducting vessels into the stem and then move into the leaf [26]. The lowest biodiversity of endophytic fungi was found in *V. amurensis* seeds, which can be explained by a better protection of the seeds by the berry pulp. Most likely, this effect was due to the fact that the berries developed later than the leaves and contained a higher number of phenolic compounds, which prevented the active endophyte accumulation. The genus *Acrospermum* was representative only for berries of wild grapevine *V. amurensis*. It has been shown that the cell wall of the fungus *Acrospermum compressum* includes unique polysaccharide containing mannofuranose. Perhaps, *Acrospermum* sp. may contain valuable substances that can later be used in biotechnology. NGS analysis revealed that endophytic fungi of the genus *Articulospora* was unique for the leaves of *V. amurensis*. As shown earlier, *Articulospora* sp. produces Art1, an inhibitor of bacterial histidine kinase [31]. Using traditional methods of cultivation of microorganisms, we identified unique strains of *Discosia* sp. for leaves of the Amur grape. This endophytic fungus exhibits promising properties for plant growth promotion. For example, the fungal inoculum *Discosia* sp. from the rhizosphere of tea significantly increased the root length, shoot length and dry matter in maize, pea and chickpea over the uninoculated control under a controlled environment [32]. Thus, these endophytic fungi of *V. amurensis* can act as a new tool for the biocontrol of certain bacteria, and can also be used in food biotechnology.

The analysis of the fungal community of *V. amurensis* collected in different years offered interesting insight into the possible correlations between fungal abundancy and whether variations. We analyzed the endophytic fungi distribution during several years (2019, 2020 and 2021). The highest numbers of fungal strains (111 strains) were detected in the cold and damp conditions in 2020 using the cultivated-dependent methods (Figure 4a, Table 1). The composition of the endophytic fungal community has also changed. The class *Tremellomycetes* became the prevalent taxa instead of *Dothideomycetes*. The main genus representing the class *Tremellomycetes* was the genus *Papiliotrema*. It is known that *Papiliotrema terrestris* [33] is a biocontrol agent isolated from the apple fruit epiphytic microbiota and selected for its ability to counteract fungal pathogens of plants and fruits both in-field and in the postharvest stages [34]. As for many biocontrol agent yeasts, the main mechanism that underlines the antagonistic activity of *P. terrestris* is the competition for nutrients and space. This mechanism relies on the ability of the biocontrol agents to rapidly colonize fruit tissues due to their adaptation and resistance to stresses generated in the wounded fruit tissues, mainly oxidative stress, as demonstrated through chemical and genetic approaches [35,36,37]. It is possible that the increase in the number of *Papiliotrema* in *V. amurensis* tissues was associated with its high adaptive potential. Thus, the average temperatures about 15 °C and a large amount of precipitation might contribute to both quantitative and qualitative biodiversity of endophytic fungi in *V. amurensis*. The hot and dry weather in 2021 could significantly reduce the number of fungal colonies.

The data obtained using the cultivation-dependent method demonstrated the seasonal variability in the composition of wild grapes mycobiome. The number and diversity of fungal endophytes was richer in autumn than in summer. In addition, a class of *Tremellomycetes* appeared in autumn and reached 47.5%. The data indicate that the fungi have not been in time to settle in the grape tissue at the beginning of the summer season compared to the autumn. The accumulation of new endophytic fungi occurs over time and when the period of leaf fall reaches its maximum value. This was probably due to the fact that endophytic fungi gradually colonized the above-ground grapevine organs, e.g., leaves, during their growth and development. The data indicate that the autumn season is the most optimal for studying the endophytic community diversity in grapevines.

The comparison of the NGS data for *V. amurensis* endophytic fungi and the previously studied fungal communities of *V. vinifera* from USA (California) showed that grapevine samples from Russia and USA located in separate clusters according to the nonmetric multidimensional scaling. The mycobiomes of grapes were represented by nine main classes of fungi. Both mycobiomes differed greatly in relative abundancies of classes. Considering some differences between the compositions of endophytic fungi of grapevines, it can be concluded that the place of growth also affected the percentage ratio between classes of endophytic fungi. At the same time, 58 genera of fungi were detected in grape samples from Vladivostok and California where 29 genera were common, despite the huge differences in the sample locations. This means that grapes preserve and maintain the necessary diversity of fungal endophytes, regardless of the place of growth. One of the underlying mechanisms could be the inheritance of endophytes with seeds or selective selection of endophytes from the environment. However, this needs further investigation with the application of additional RNA-seq libraries from other cultivars.

Taken together, the obtained data can be used to create potential vineyard biocontrol agents for plant pathogen protection. Thus, future studies on the biochemical properties (e.g., the ability to secrete phytohormones or biologically active substances) or biological functions (e.g., plant disease protection) of isolated endophytic fungi can greatly contribute to crop protection and plant functional studies.

## 4. Materials and Methods

### 4.1. Plant Material

This study used tissues of two healthy 10–15-year-old vines of *V. amurensis* located at a distance of 1 km from each other in a nonprotected natural population near Vladivostok, Russia (the southern Primorsky Territory of the Russian Far East, longitude 43.2242327 and latitude 131.99112300). Shoots, leaves (young stems 7–8 cm long with three healthy leaves), berries (green and mature), and seeds were collected at 10–11 a.m. on low-cloud days without precipitation, the air temperature was 17–20 °C. The values of the average temperatures and precipitation in 2019–2021 in Vladivostok (Primorsky Territory of Russia) are shown in the Table 1. Each plant material specimen was delivered to the laboratory within 30 min.

For the cultivation-dependent approach (fungal sowing), the plant material was collected in July and September from 2019 to 2021. Two biological replicates (two individual vines) were collected in July, and two biological replicates—in September. Thus, there were four biological replicates per year. In total, 12 biological replicates were collected and analyzed by the cultivation-dependent approach from 2019 to 2021. For the cultivation-independent approach (NGS), we used grapevine material collected in July and September of 2021 (a total of 4 biological replicates) and applied 2 technical replications per biological replicate. 

### 4.2. Isolation and Identification of the Endophytic Fungi

The grapevine tissues (1.5 g) were washed under running water with soap and washed sequentially under sterile conditions in 75% ethanol for 2 min, 10% hydrogen peroxide for 1 min, and five times in sterile water. To check the efficacy of this method of surface sterilization, 100 µL of the last wash water was incubated on potato–dextrose agar medium (PDA, Neogene, UK). No microorganism growth was observed 7 days after the last portion of washing water had been plated in the Petri plates containing the growth media. This validated the quality of the performed superficial sterilization of the grape tissues.

The surface-sterilized tissue of *V. amurensis* was ground to a homogeneous mass in a sterile mortar; the resulting juice was squeezed, and a 100 μL aliquot was transferred to PDA plates. After 7 days, the grown fungi colonies were sampled and carefully transferred to a new sterile Petri plate for repeated cultivation. We isolated almost all the seeded isolates into separate strains, a total of 199 separate strains of endophytic fungi were obtained over 3 years of biological isolation technique.

DNA of the 199 fungi strains was isolated by the hexadecyltrimethylammonium bromide (CTAB) method with modifications [38]. Fungal *ITS1* rDNA were amplified using universal primers for the amplification of approximately 560 bp *ITS1* PCR products (5′AGG AGA AGT CGT AAC AAG G and 5′TCC TCC GCT TAT TGA TAT GC) [39]. PCR products were sequenced using an ABI 3130 Genetic Analyzer (Applied Biosystems, Foster City, CA, USA) according to the manufacturer’s instructions as described [18,40]. The Basic Local Alignment Search Tool (BLAST) program was used for sequence analysis. Multiple sequence alignments were performed using the Clustal X program [41]. A sequence identity of ≥99% was considered as a sufficient threshold value for taxonomic identification of bacteria genus.

### 4.3. DNA Extraction, PCR Condition, Library Preparation, and Sequencing

DNA for NGS was isolated using two approaches. The first one used the method used in our lab with small modifications [42]. According to the second approach, the DNA was extracted from 50 mg of *V. amurensis* tissue using the ZymoBIOMICS DNA miniprep kit per manufacturer’s protocol (Zymo Research, Irvine, CA, USA). DNA was assessed for quality and quantity using the NanoPhotometer P300 (IMPLEN, Munich, Germany). 

The DNA samples were sent to Evrogene (Moscow, Russia) for high-throughput sequencing using Illumina technology. The libraries were prepared for sequencing according to the protocol described in the manual “16S Meta-genomic Sequencing Library Preparation” (Part # 15,044,223 Rev. B; Illumina). Fungal *ITS1* rDNA regions were amplified from all samples using primers ITS1f (5ꞌCTTGGTCATTTAGAGGAAGTAA) and ITS2 (5ꞌ GCTGCGTTCTTCATCGATGC) [26]. After obtaining the amplicons, the libraries were purified and mixed equimolarly using the SequalPrep™ Normalization Plate Kit (ThermoFisher, Cat # A10510-01). Quality control of the obtained library pools was performed using Fragment Analyzer and quantitative analysis was performed using qPCR. The library pool was sequenced on Illumina MiSeq (2 × 250 paired end) using MiSeq Reagent Kit v2 (500 cycles). The FASTQ files were obtained using bcl2fastq v2.17.1.14 Conversion Software (Illumina). The phage PhiX library was used to control sequencing parameters. Most of the reads pertaining to phage DNA were removed during demultiplexing.

Fungal sequences were deposited in NCBI under the accession number PRJNA874841 and in the database of laboratory Biotechnology, Federal Scientific Center of the East Asia Terrestrial Biodiversity, Far Eastern Branch of the Russian Academy of Sciences, Russia (https://biosoil.ru/downloads/biotech/Vitis%20metagenom/2021-09=Vitis_amurensis_endophytes_ITS), (accessed on 5 October 2022).

### 4.4. Computational Analysis

The samples used in the bioinformatic analysis are presented in Supporting Information 7. Raw readings were preprocessed using QIIME 2 [43] and DADA2 programs [44]. The primers, remaining PhiX reads, and chimeric sequences were removed, and paired-end reads were merged and sorted. Taxonomic identification of sequences was performed using the QIIME 2 Scikit-learn algorithm [45] using the UNITE pre-trained classifier (99% OTUs from ITS1f/ITS2 region of sequences) [46].

The obtained data were processed using the R language. The phyloseq library [47] and tidyverse package [48] were used in pre-filtering and data preparation. Taxa for bar plot, heatmap and UpSet diagram visualizations were filtered based on relative abundance of >0.1% for each biocompartment. In bar plots, we merged the taxonomic ranks that were relatively abundant < 0.1% in each factor to one group called “other”. Shannon alpha diversity and Bray–Curtis beta diversity data were obtained using the Vegan package [49]. Bray–Curtis dissimilarity data were transformed to even sampling depth and converted to nonmetric multidimensional scaling (NMDS). A Wilcoxon rank sum test was performed to analyze the alpha diversity data between groups. Statistical validation of beta diversity data was performed using the PERMANOVA test with 999 permutations [49]. The ggplot2 [48] and ComplexHeatmap [50] R libraries were used in the graphical representation of the results.

## Figures and Tables

**Table 1:** The values of the average temperature and precipitation in 2019–2021 in Vladivostok, Primorsky Territory of Russia.

Summer	Average t, °C	Precipitation, mm	Autumn	Average t, °C	Precipitation, mm
The Norm	18.1	159	The Norm	16	103
July 2019	17.1	131	September 2019	17.3	44
July 2020	14.7	281	September 2020	16.3	138
July 2021	21.3	24	September 2021	17.7	120

http://www.pogodaiklimat.ru/ (accessed on 14 June 2022).

## Data Availability

The data presented in this study are available within the article and Supplementary Material.

## Supplementary Materials

The following supporting information can be downloaded at: https://www.mdpi.com/article/10.3390/plants11212897/s1, Supporting Information 1. Intersections in our metagenome data (Organ material)—Figure S1c; Supporting Information 2. NMDS ordination plots and PERMANOVA results in our data; Supporting Information 3 Intersections in our data (Data type)—Figure S2; Supporting Information 4. Intersections in data of cultivate-dependent method (Date)—Figure S4b; Supporting Information 5. Intersections in data of cultivate-dependent method (Season)—Figure S5c; Supporting Information 6. Intersections in our metagenome data (Season)—Figure S5d; Supporting Information 7. Samples used in comparative analysis of the grape mycobiomes.
